# Valuing education for limited-resource audiences using a willingness to accept experiment

**DOI:** 10.1371/journal.pone.0348858

**Published:** 2026-09-16

**Authors:** Russell Tronstad, Ibrahima Sall

**Affiliations:** Department of Agricultural and Applied Economics, The University of Arizona, Tucson, Arizona, United States of America; National Chung Hsing University, TAIWAN

## Abstract

Education grants often target audiences that have limited-resources relative to their peers, such as beginning farmers, who arguably have the most to gain from more education. Yet, charging even modest fees to these participants is discouraged, creating a challenge for funding entities that desire to have a dollar value for the education provided. To measure this value, we conducted an experiment using a willingness-to-accept (WTA) approach. Participants chose a minimum cash payout from seven different options to voluntarily leave a training session before receiving any instruction or educational materials. The design is constructed so that each option has an equal expected payout. Cash payouts were not advertised or known to any participants before the training. Results show that average WTA values are $277.44 per training (about 4 hours of instruction), which is 2.11 to 4.74 times more than participants’ self-reported willingness to pay (WTP), depending on how travel costs and time of participants are valued. WTP consistently and significantly explains much of the variation in WTA. In sharp contrast, detailed travel cost estimates in various forms were consistently insignificant for explaining WTA values. Beginning farmers and ranchers, our targeted limited-resource audience, reported WTA and WTP values that were $81.18 and $22.73 more per training, respectively, than those of non-beginning farmers.

## 1. Introduction

Measuring the impact of educational programs in monetary terms is a longstanding challenge. Program evaluators often rely on participants’ self-reported change in knowledge before and after receiving education, or even pre- and post-education quizzes, to quantify the value of education [1,2], but these changes in knowledge do not translate into a dollar value that can be provided to funding agencies. Public agencies and non-profit organizations that fund education are interested in assigning a dollar value to the benefits of the education they support. The amount participants pay in registration fees yields a quantifiable dollar value for education. However, individuals with relatively limited resources and little experience are generally unable to pay high registration fees, even though they arguably have the most to gain from additional education. This makes traditional evaluations, including willingness to pay (WTP) assessments, challenging for valuing these programs.

To address this issue, we propose an experimental approach that uses willingness to accept (WTA) as an alternative method to evaluate the value of educational programs. WTA reflects the minimum compensation someone requires to give up a good or service, such as skipping a training session they would normally attend. Economic theory and a large empirical literature confirm that WTA typically exceeds WTP, for reasons that include loss aversion and, importantly here, the fact that WTP is limited by a person’s budget or ability to pay, while WTA is not [3–5]. For example, a participant with limited income might only be able or willing to pay a small fee but still insist on a larger compensation to give up the opportunity. We therefore expect participants’ WTA for a training session to exceed their WTP, especially among resource-limited participants. Section II develops this background in more detail.

Travel cost has been widely used in non-market valuation studies [6–9]. Parsons [8] outlines how the travel cost model should include transportation, access, equipment, and time costs. We account for differences in WTA values by considering transportation and time costs. Travel costs are already incurred and sunk for participants when we unknowingly solicit their WTA value for a training session they have already traveled to. However, the maximum WTP amount participants provide for a training session would be their total expected educational benefit less transportation and time costs.

Our study focuses on beginning farmers and ranchers (crop or livestock focused producers) in the Southwest of the United States as the primary audience. This demographic exemplifies newcomers who generally operate small-scale farms with minimal capital, hence relatively limited-resource producers, and a need for education. We conducted an experiment integrated into federally supported training sessions designed for beginning farmers and ranchers. Each session was approximately six hours, including lunch and breaks, and focused on practical agricultural topics. Supported by an educational grant, the programs were held in person at little or no cost to participants, with a small $10 registration fee that mostly covered lunch. The setting and recruitment methods reflected typical extension education programs, in which participants were recruited through cooperative extension and partner networks and voluntarily enrolled. A total of 146 producers attended beginning farmer and rancher education at these two rural locations in the Southwest US; 118 voluntarily returned questionnaires, of which 114 were sufficiently complete for analysis. Since these initiatives aimed to enhance the skills and success of new farmers, the values gathered from participants highlight the significance they place on such educational opportunities.

The primary objective of this study is to present and apply a new experimental valuation methodology to more accurately value education for participants who are relatively resource-constrained (i.e., beginning farmers and ranchers in this study), by focusing on their WTA compensation for not attending training. The remainder of the paper proceeds as follows. Section II reviews the related literature and conceptual framework. Section III explains the design and implementation of the experiment, along with data collection methods. Section IV lays out a comprehensive framework for estimating travel costs and the modeling steps. Section V presents our results, including both descriptive data and regression analyses that link WTA to WTP and other variables; we especially focus on the difference between WTA and WTP and what it suggests about participants’ valuation of the training. We analyze these findings from both theoretical and practical perspectives and discuss the limitations of our approach, such as potential hypothetical bias in WTP and biases associated with the WTA method, to keep our conclusions balanced. Finally, Section VI provides final thoughts on the value of the WTA method for evaluating programs and policies, suggesting cautious yet hopeful implications for funding educational initiatives for those who need them most.

## 2. Literature review and conceptual background

A large body of valuation research shows that the compensation people demand to give up a good (WTA) generally exceeds what they are willing to pay to acquire the same good (WTP). Hanemann [4] demonstrated that, beyond measurement errors, a genuine WTA–WTP gap can arise from income and substitution effects, with the gap widening as substitutes become scarcer. Reviewing the empirical evidence, Horowitz and McConnell [3] found mean WTA/WTP ratios often approaching seven, while the more recent meta-analysis by Tunçel and Hammitt [10] reported an average ratio around three across goods and above six for environmental goods. Two patterns from that meta-analysis are directly relevant to our design: the disparity is smaller when respondents have market or repeated experience with the good, and smaller when values are elicited through incentive-compatible mechanisms rather than hypothetical questions. Frondel, Sommer, and Tomberg [11] similarly reported ratios of 2.35–3.56 and emphasized that the perceived realism of the valuation setting influences the size of the gap.

The leading behavioral explanation attributes the gap to the endowment effect and loss aversion: once people feel ownership of a good, parting with it is perceived as a loss and is weighed more heavily than an equivalent gain [5,12]. However, Plott and Zeiler [13] showed that a significant part of the observed gap can be “turned on and off” through experimental procedures that address subject misconceptions about the elicitation device, cautioning that an observed WTA–WTP gap alone does not prove loss aversion. This literature motivates two design choices in our study: using real monetary stakes instead of hypothetical questions, and providing participants with a clear, practiced elicitation process.

A mechanism is incentive-compatible when truthful revelation of one’s value is a (weakly) dominant strategy. The canonical single-response device is the Becker–DeGroot–Marschak (BDM) mechanism [14], in which an individual states a value and a randomly drawn price determines whether a transaction occurs at that price, so that misstating one’s value can only make the individual worse off. Related logic underpins experimental auctions, which substitute real exchange for hypothetical questions to recover demand-revealing valuations [15], and Carson and Groves [16] extend incentive-compatibility reasoning to stated-preference and contingent-valuation questions, clarifying the conditions under which survey responses can be consequential and truth-revealing. Recent applied work continues to deploy these tools in agricultural settings: Cole and Fernando [17] use the BDM mechanism to elicit farmers’ WTP for a mobile agricultural advisory service in India, and Mangole et al. [18] use a BDM experiment to elicit smallholder farmers’ WTP for a digital extension service in Tanzania and Burkina Faso.

Research valuing training and advisory programs often categorizes explanatory variables into program attributes (fee, topic, duration, and certification), participation costs (distance, travel time, and opportunity cost), individual characteristics (age, education, experience, and income), institutional and trust factors (confidence in the provider or the sponsoring institution), and behavioral or risk factors (risk and time preferences, and perceived benefit or efficacy). The first three groups are commonly used in contingent-valuation and choice-experiment models of extension demand. In contrast, measures of institutional trust and behavioral risk are less consistently collected, although institutional variables are among the drivers of stated WTP where they are gathered [18]. These factors can influence both participation and stated value. Trust in the advisor or the sponsoring institution affects farmers’ engagement with advice [19], and risk attitudes, which can be measured with established tools and differ widely among farmers [20], or perceived benefit can influence outcomes. Our design directly captures program attributes, participation costs, and individual characteristics. Although we did not administer validated trust or risk-preference instruments, we recorded two perception items, perceived benefit of the session and its impact on the participant’s future direction, which we use as sensitivity checks on the main models (Section IV.2.3).

Our elicitation technique reflects the incentive-compatible approach of BDM and experimental auctions while adapting it to a field education setting. Participants select the lowest of seven cash amounts they would accept to leave a training session before any instruction, real payouts are decided by a two-stage lottery, and the number of tickets is inversely related to the dollar amount so that the expected payout of each option is equal by design. Because higher stated amounts are drawn less frequently, the setup discourages overstatement by lowering the chance of receiving a higher payment, while understating risks being required to leave the session for less than it is worth to the participant, features intended to encourage truthful revelation, similar to the spirit of BDM. However, it is not the classic BDM procedure: the choice set is discrete rather than continuous, the actual payment is determined through a lottery over a fixed prize pool rather than a continuously drawn buying/selling price, and the object being valued is a service the participant has already traveled to attend. We therefore describe our design experiment as practical to implement for approximating incentive compatibility in the field rather than as a strict BDM auction.

Economic evaluation of educational and extension programs has typically relied on knowledge gains, productivity or adoption outcomes, or registration fees and WTP. A growing recent literature elicits farmers’ WTP for extension and advisory services directly [17,18,21]. A recurring theme is that stated WTP for such services is strongly conditioned by income and ability to pay: in Ghana, demand for a digital agriculture and nutrition service fell steeply as its price rose [21], and in India, BDM-elicited WTP for a mobile advisory service, although positive, fell short of the service’s per-farmer cost at the scale studied [17]. This is precisely the difficulty for the limited-resource audiences in our study, beginning farmers and ranchers. Their WTP is constrained by tight budgets even when the underlying value of education is high, such that WTP-based evaluations risk understating the value of programs for the very groups they are designed to serve.

Because WTA measures the compensation required to forgo a good and is not capped by a participant’s budget, it is well suited to valuing education for resource-constrained learners, for whom the income constraint binds most tightly [4,21]. In our setting, participants have already incurred (sunk) travel costs to attend, so a WTA elicited at the door may capture value that an income-limited WTP measure would understate. Stated WTA can also reflect opportunity costs, expectations about the session, sunk-cost or context effects, and may be inflated by loss aversion or by misunderstanding of the mechanism [5,13]. We therefore interpret WTA as an informative complement to WTP rather than a clean measure of instructional value alone, reporting both and treating the WTA–WTP gap as evidence on how income constraints and perceived value jointly shape stated values.

## 3. Experimental design and data collection

To better quantify the value of education to participants, we utilize elements of a field experiment by soliciting participants in their natural environment with choices that have economic consequences. That is, cash is exchanged for education that participants travel to attend as they normally would, if their ticket is drawn. As participants arrive before a training session, they are provided a questionnaire and one side of a double ticket. The blank instrument is provided as S1 Appendix. They are then asked to select what is the minimum cash value, among the seven amounts of $25, $50, $71.5, $100, $125, $250, and $500, that would be required for them to voluntarily leave before the scheduled training starts without also receiving any educational materials.

**Fig 1:**
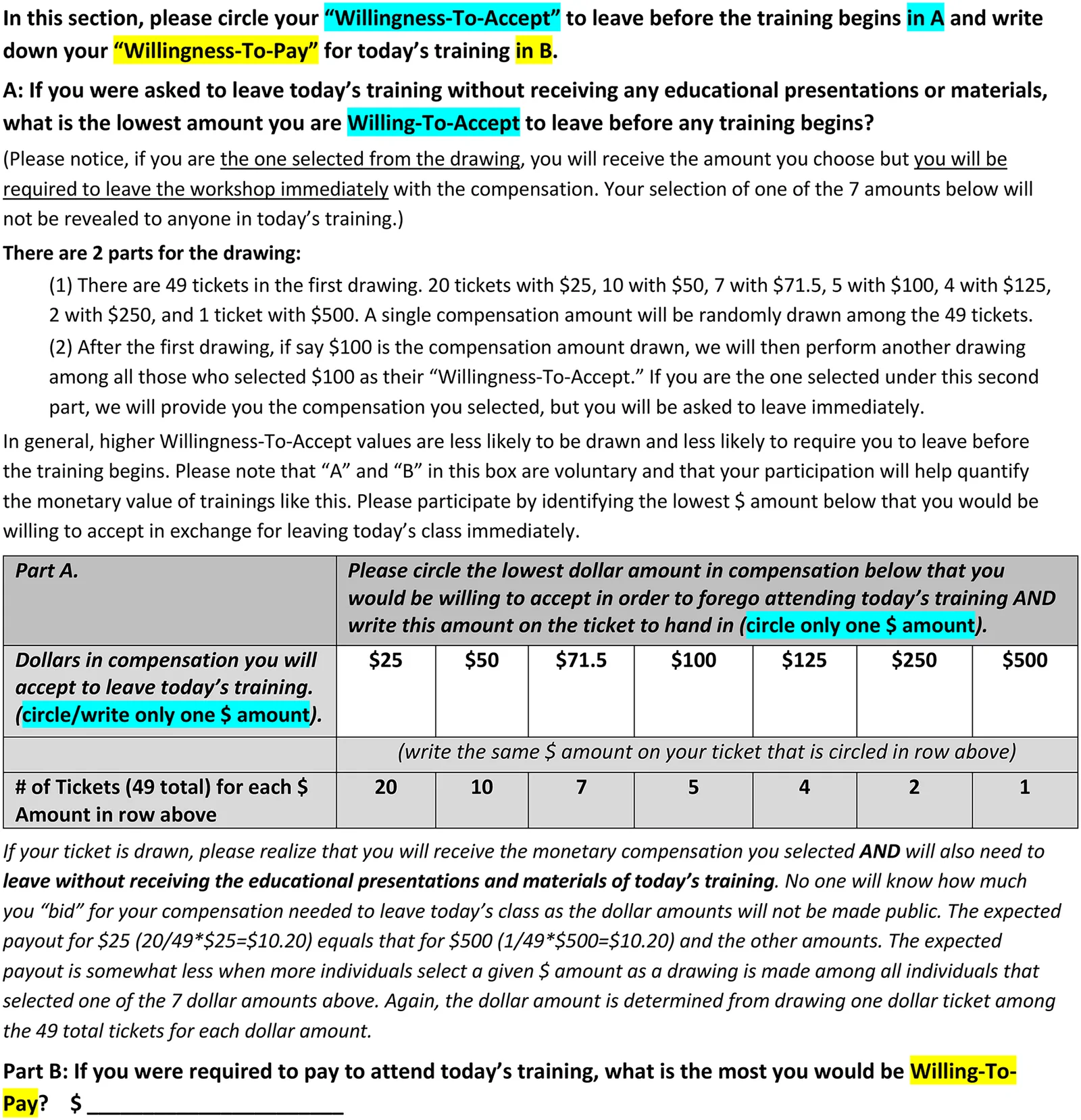
Written explanation provided to participants on their willingness to accept and willingness to pay for the training provided.

Oral directives combined with the written instructions in Fig 1 are then provided to clarify that two sets of drawings will take place. Participants were informed that two drawings would take place:

- *Selection of the Offer amount*: A drawing is conducted among 49 tickets (20 of $25, 10 of $50, 7 of $71.5, 5 of $100, 4 of $125, 2 of $250, and 1 of $500) to determine the dollar amount of cash that will be awarded. The distribution of tickets was inversely proportional the dollar amount (e.g., 20 tickets for $25, 1 ticket for $500) (Fig 1) so that the expected value associated with each of the seven WTA values is exactly $10.20.
- *Selection of the Winner***:** If a specific dollar amount was drawn (e.g., $100), a second drawing is conducted among all tickets provided by participants with WTA values equal to the first drawn dollar amount to determine which ticket holder or participant will have the opportunity to receive the cash amount they identified that will adequately compensate them for leaving before any education is provided.

This design aimed to balance the expected payouts across options in advance, encouraging participants to choose based on their personal valuations rather than just the highest expected value. Participants are informed that higher WTA values are less likely to be drawn and that no other participant will know the amount they select for their WTA, as the dollar amount drawn in the first drawing is not revealed to others.

Participants are then asked to write their WTA value on both their questionnaire and double ticket. At this time, they are also asked to identify their maximum WTP to attend today’s training on the questionnaire. After participants write their WTA value on one side of their double ticket with the dollar sign, their tickets are collected in a container. Participants are then instructed to answer other questions about their demographics and how they traveled to the event, while refraining from answering questions about the actual knowledge gained during the training. The questions at the end ask for their self-reported understanding of the subjects covered before and after the training, and how they feel the education provided will affect their future success and direction. Data were collected at two different locations where beginning farmer or rancher oriented education was provided. All participants were solicited to complete the questionnaire even though completed responses from all questions were not obtained. Definitions and summary statistics for the variables used in our analysis are provided in Table 1.

**Table 1:** Definitions and descriptive statistics of variables.

Variable Name	Variable Description	Mean	Median	N
*WTA*	The lowest of the seven dollar values ($25, $50, $71.5, $100, $125, $250, and $500) presented that a participant is willing to accept to leave with no educational materials before the scheduled in-person training begins.	$277.44	$250	94
*WTP*	What each participant indicates they are willing to pay to attend the scheduled in-person training (~ 4 hours of education) before any education starts.	$58.47	$50	73
*Npeople*	Number of participants that traveled together, including the respondent.	1.86	2	113
*Nmiles*	Number of miles (one-way) to attend training.	88.92	55	114
*TravC*	Estimated travel cost defined as: TravC=Cost of transport+Value of traveltime $Cost\;of\;transport=\frac{(2\times Nmiles)\times Costpermile}{Npeople}$Value of traveltime=(2×Nmiles×AvgMPH)*(Wage/3) *AvgMPH* = average miles per hour driving speed from *k* distant points participants travel to a training location. *Wage* = hourly wage as a function of education and gender	$69.08	$42.25	107
*OPCattend*	Estimated opportunity cost of total time spent (~ 6 hours for training, breaks, lunch, and transitions) at the site Cattend=6*(Wage/3)	$41.03	$40.84	107
*TotC*	*TCost = TravC + OPCattend*	$110.11	$83.06	107
*WTA* – *TravC*		$208.75	$181.41	90
*WTP + TravC*		$131.21	$100.43	70
*DBegFarm*	Dummy variable for beginning farmer (*DBegFarm* = 0 if farming/ranching for more than 10 years; else = 1).	0.66	1	114
*AvgKnowBef*	Average understanding or level of knowledge on topics addressed of before attending the training. Scaled from 1 to 5 with 1 = Poor and 5 = Excellent.	2.20	2	105
*AvgKnowAft*	Average understanding or level of knowledge on topics addressed after attending the training. Scaled from 1 to 5 with 1 = Poor and 5 = Excellent.	3.67	4	100
*Education*	The number of education years completed.	14.87	14	108
*Male*	Dummy variable for gender (Male = 1 if the gender is male and 0 if female)	0.52	1	113
*Age*	Age of the participant	48.5	49.5	114

## 4. Methods

This study was reviewed and approved as exempt human-subjects research by the University of Arizona Institutional Review Board. All participants were adults 18 years or older, with no minors involved. Prior to participation, attendees received an IRB-approved verbal informed-consent script that explained the study’s purpose, the voluntary nature of participation, the right to decline or stop at any time without penalty, and the confidentiality of responses. The IRB approved a waiver of informed consent documentation, so no signed consent forms were collected; consent was indicated by voluntarily completing the anonymous questionnaire. No direct identifiers were collected.

### 4.1. Travel cost

We first use self-reported distances traveled to determine transport costs and then use the Google API and a Spillman function to estimate travel duration. Next, we add the opportunity cost of travel time using estimated hourly wages to determine total travel costs.

#### 4.1.1. Value of travel time.

Travel time cost, often an overlooked component in survey-based studies, carries significant weight when determining the non-market value of resources or services. Traditionally, estimating travel time cost involves methodologies, relying heavily on broad averages [6]. While studies have widely acknowledged the opportunity costs associated with travel time, a predominant challenge lies in estimating the average speed traveled of individual respondents [22]. This challenge stems from many variable factors influencing travel speed, including route, traffic conditions, and individual driving behaviors. Our research seeks to address this complexity by leveraging advanced geospatial technologies to produce more defensible estimates of expected individual travel speeds from respondents’ self-reported travel miles. Through the application of isochrones or contours representing equal travel time for a given distance, we derive expected travel duration times [23] using Google’s Directions API and the self-reported travel distances from each training location. When measuring the opportunity cost of travel time, we prefer to use expected travel time rather than actual travel time. This is because the distance used to calculate expected travel time remains constant, while actual travel time can vary dramatically and unexpectedly due to factors such as accidents, general traffic, and road conditions. Therefore, expected travel time is believed to be a relatively accurate way to determine the anticipated costs of travel time for individuals.

**Fig 2:**
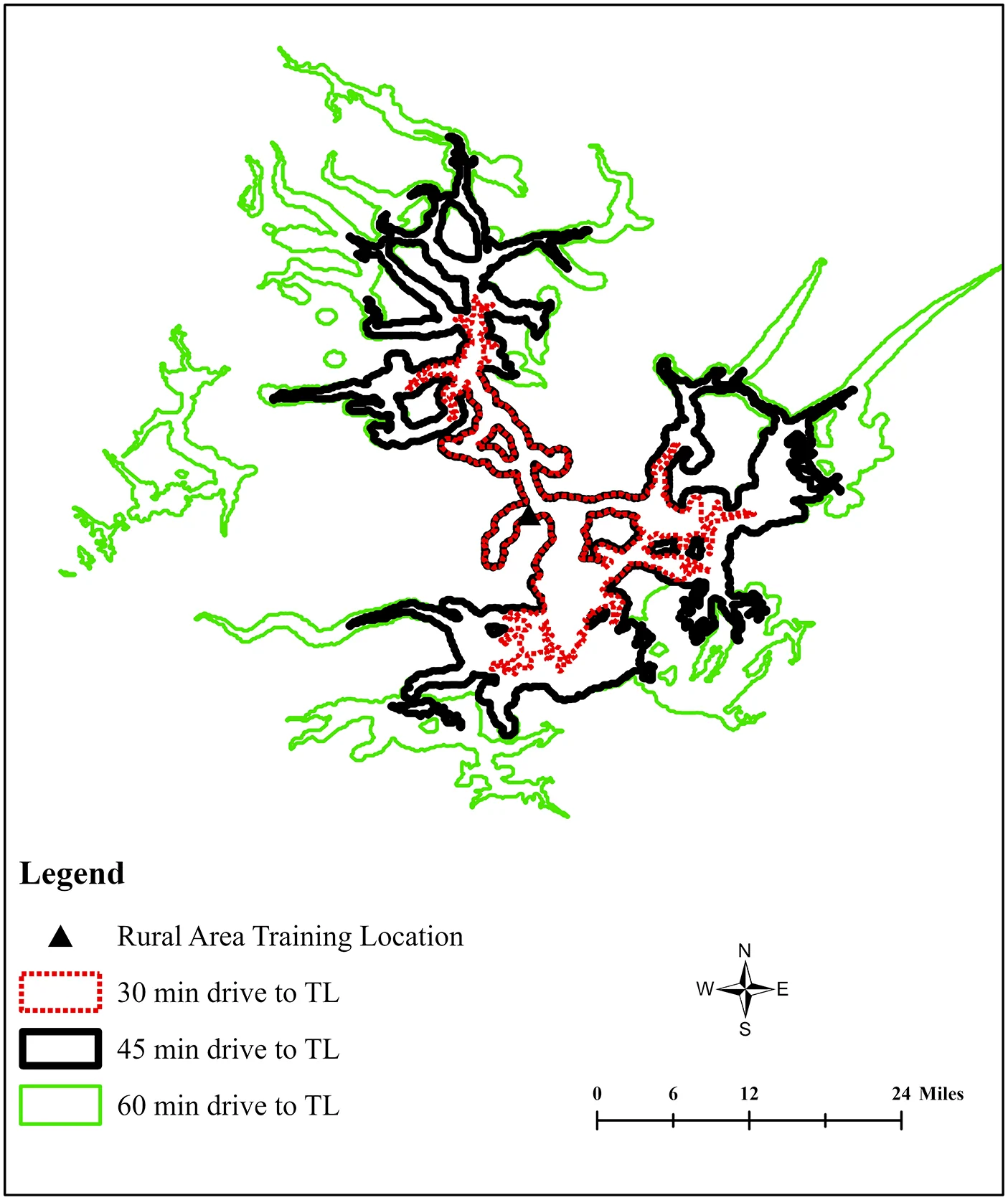
Isochrones for training location that was 47 miles away from nearest town (<17K population). Author-generated drive-time isochrone figure on a blank background; no proprietary basemaps, satellite imagery, street maps, topographic maps, or map tiles are reproduced.

**Fig 3:**
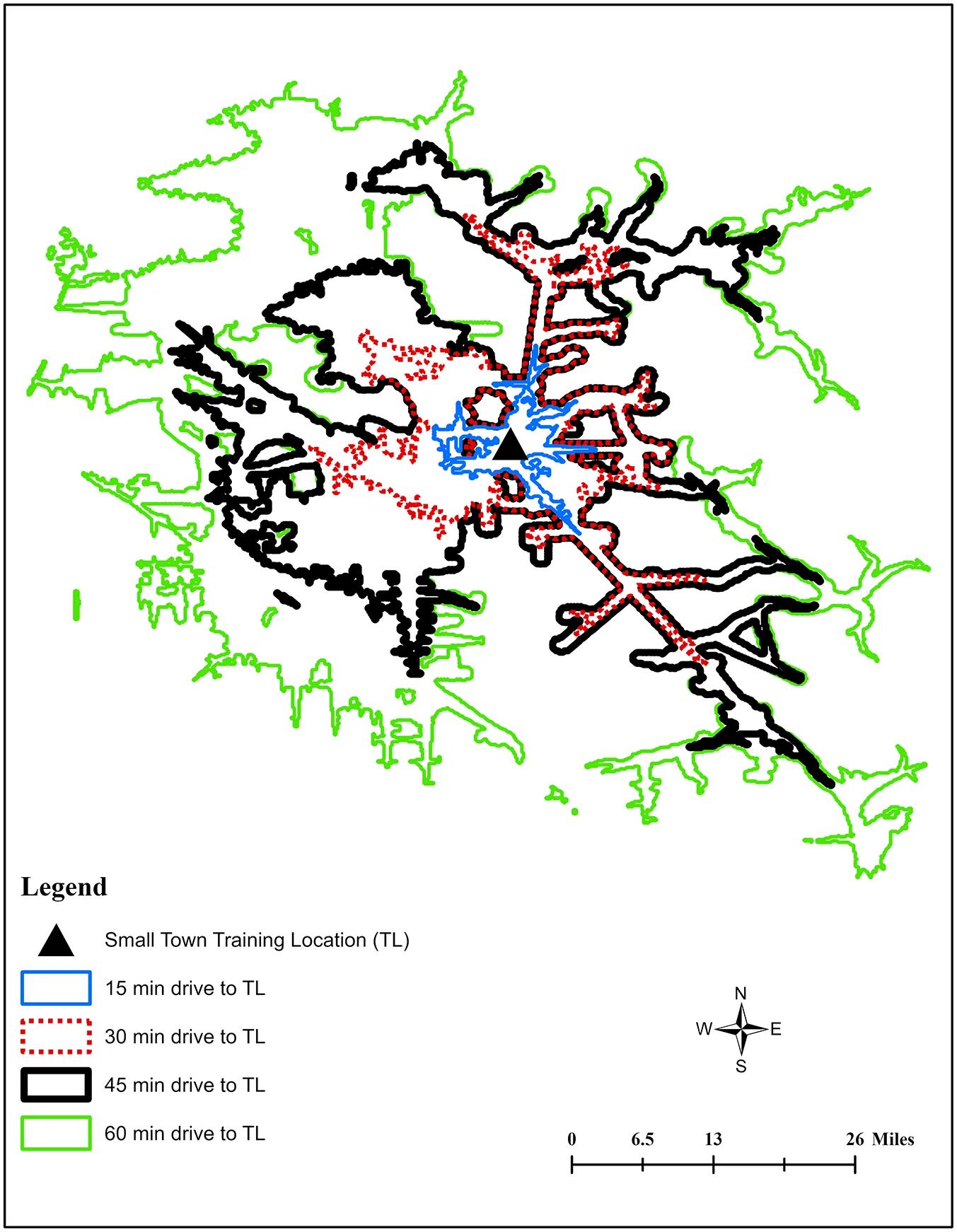
Isochrones for training location that was in small town (<3K population). Author-generated drive-time isochrone figure on a blank background; no proprietary basemaps, satellite imagery, street maps, topographic maps, or map tiles are reproduced.

Isochrones are like contour lines which show areas accessible within the same duration of travel time from a particular point (Figs 2 and 3). We utilize Open Source Routing Machine (OSRM), an efficient open-source solution known for its routing algorithms, to generate these isochrones [24]. One training location was in a small town with a population of less than 8,500, and another was in a somewhat remote area that is 47 miles away from the nearest population of just over 11,000. OSRM calculates isodistances (equal distance contours) and isochrones (equal travel time contours) by simulating travel times across various routes. It takes into account factors such as road types, speed limits, and turn restrictions to estimate how far one can travel within a given timeframe, creating a spatial boundary that reflects realistic accessibilities.

After generating isochrones, we utilize the Googleway package [25] to access Google’s API Directions to determine travel times and distances from the isochronally-generated points to our study locations. The Google Directions function within Google Maps inputs the derived geographic coordinates (latitude and longitude) and computes optimal travel paths based on Google Maps’ extensive repository of traffic patterns, road network data, and legal travel speeds. Google Directions API was used only to obtain numeric travel-time and distance estimates for the travel-cost calculations; no Google map image, basemap, satellite image, or map tile is reproduced in any figure in this article.

It is possible that participants may not keep track of their travel time as accurately as they would the mileage from their location of origin due to variability in factors such as traffic and road conditions. To overcome this limitation, we present an estimation method for the expected travel time of each participant. To assign an actual travel time for a participant’s self-reported distance traveled to a training location, we utilize the Spillman functional form to provide a point estimate among numerous possible locations that are possible. The Spillman functional form, typically employed in agricultural production studies to describe the yield response to variable inputs, is characterized by incorporating diminishing but never negative returns to scale. In the case of travel speed, it utilizes an exponential term that effectively models the saturation point of the speed limit, beyond which increases in distance result in proportionally smaller increments in speed as average speed never declines for higher travel distances. This is particularly relevant for travel in rural areas, where the acceleration in speed is likely to plateau due to factors such as road quality, adherence to safety norms, and lower traffic density, as opposed to urban scenarios characterized by frequent stops and congestions [26].

We adapt the following Spillman functional form [27,28]:


$$\begin{array}{c} AvgMPH_{k}=M-A•R^{Distance_{k}} \end{array}$$ (1)

where *M*, *A*, and *R* are parameters to be estimated, *AvgMPH* is the estimated average speed in miles per hour, and *Distance* is the miles from a training location to *k* distance points traveled by participants.

**Fig 4:**
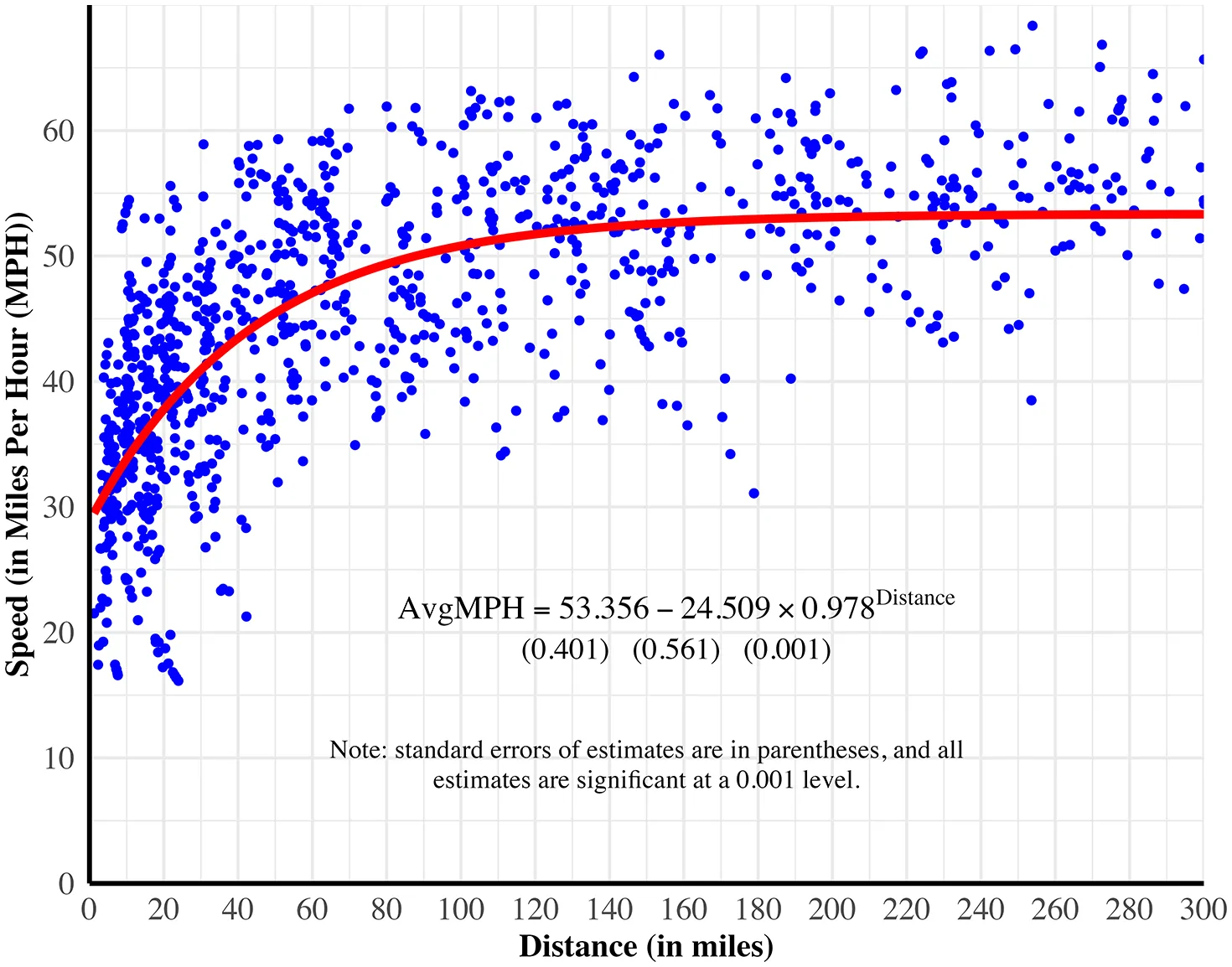
Speed points generated from isochrones and estimated average speed using the Spillman function for a rural area training location held near a small rural town (<3K population).

**Fig 5:**
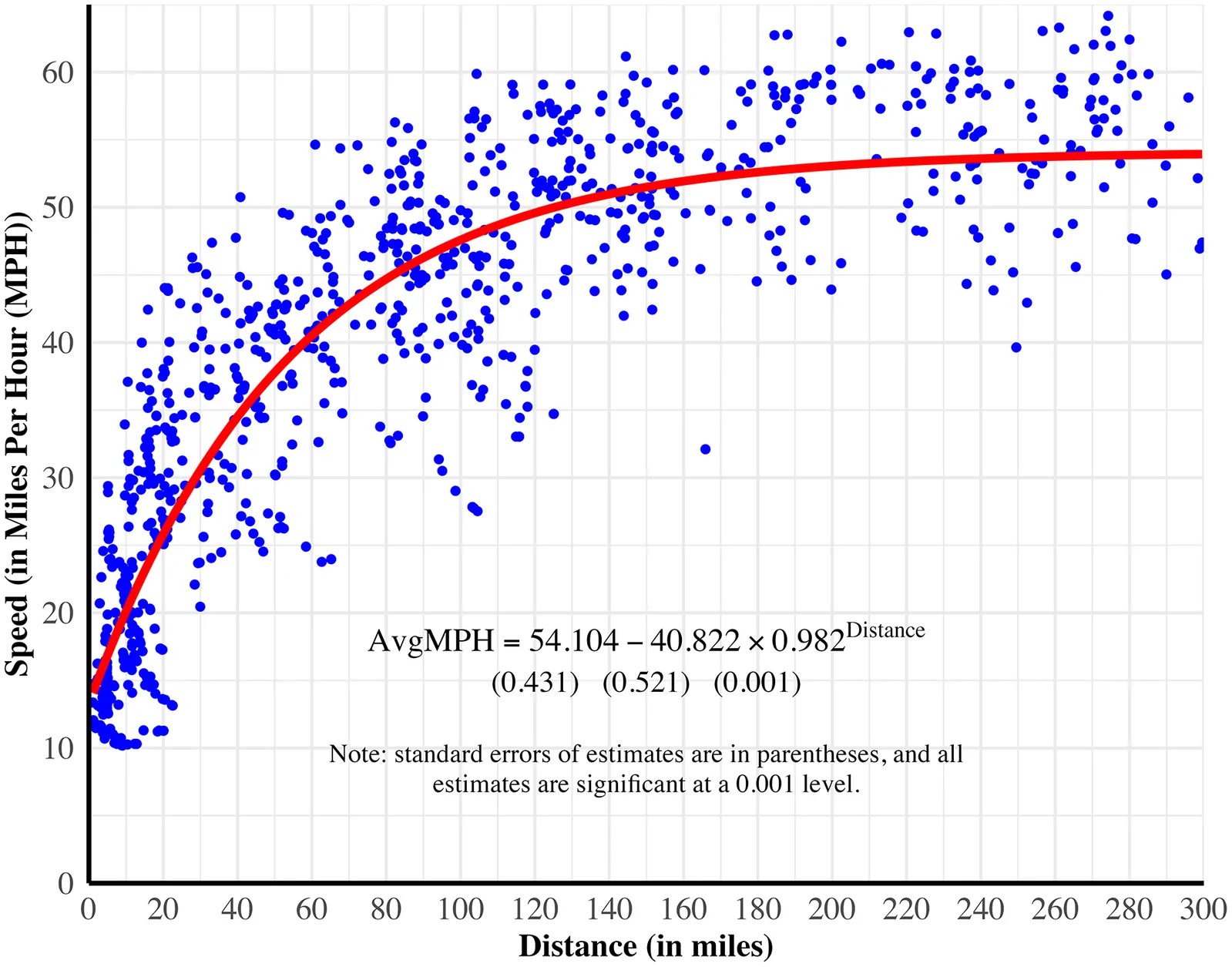
Speed points generated from isochrones and estimated average speed using the Spillman function for a small town training location 47 miles away from the nearest town (<17K Population).

To estimate the parameters of our Spillman function, we utilize simulated Google API average speeds from each training location. Then we use our estimated Spillman relationship between average speed and distance to determine each participant’s average speed and time spent. The estimation process involves identifying initial values for parameters *M*, *A*, and *R*, informed by the observed data range and theoretical considerations. Specifically, *M* was set to a value slightly above the maximum observed speed to account for potential unobserved optimal conditions, while A was initialized as the range of observed speeds, and *R* was tentatively placed at a midpoint value (see Figs 4 and 5).

Given that our data comprised only the distance from the training venue and a participant’s travel origin, the framework provides a means to incorporate diminishing returns without requiring additional information on traffic conditions or individual driving behavior. This approach represents an improvement over conventional constant-speed methods [6]. Furthermore, the Spillman functional form, offers a defendable alternative for travel cost estimation by accounting for the unique characteristics of rural travel, and enhancing the precision of travel time cost valuation in non-market resource evaluation [8].

#### 4.1.2. Total travel costs.

We account for hourly wages, travel times, transportation costs, and the opportunity cost of time for attendance to determine total travel costs. Stata and R software are used for data manipulation and analysis. To maintain analytical consistency, we set travel costs equal to zero for the few whose travel was entirely financed by their employer or organization, indicating they had no direct out-of-pocket costs or opportunity costs for their travel and attendance time, as those costs were covered by their employer.

The cost of travel is calculated using the American Automobile Association (AAA) standard rate corresponding to the estimated cost per mile for 15,000 miles driven per year adjusted by the number of individuals sharing travel expenses. The training sessions analyzed were conducted in 2019, 2020, and 2021. We calculate the opportunity cost of time using the wages from “State of Working America Wages” [29]. Similar to other studies [6,7,9], the hourly opportunity cost of travel time was set to be one-third of the hourly wage rate earned by each classified individual’s characteristics. However, in this study, we use the median hourly wage rather than the average hourly wage.

### 4.2. Regression analyses

To examine the factors influencing participants’ WTA, we initially applied Ordinary Least Squares (OLS) regression, considering the recorded WTA as a continuous dependent variable. Although WTA is fundamentally an interval-censored variable with seven distinct values, beginning with OLS provides a simple baseline and helps interpret linear relationships. We developed multiple OLS models to analyze how WTA relates to willingness to pay (WTP) and travel costs. To account for potential heteroskedasticity (unequal error variance) in the cross-sectional data, we used robust standard errors in all models [30,31]. Employing heteroskedasticity-consistent standard errors ensures the reliability of our inferences, even if WTA responses exhibit greater variability among individuals. We also assessed the assumptions regarding the distribution of residuals. A Shapiro–Wilk test [32] on the OLS residuals strongly rejected the null hypothesis of normality (p < 0.001 for all specifications), suggesting that the residuals are likely non-normal due to the discrete and skewed nature of WTA. This diagnostic further validated our choice to report robust standard errors and to later explore an ordinal model that is more appropriate for the seven categorical WTA outcomes.

#### 4.2.1. OLS specification.

We developed a series of ordinary least squares (OLS) models with the dependent variable being the stated willingness to accept (WTA) amount for participant *i*. The main independent variables include the participant’s own willingness to pay (WTP) for the training and their travel cost measures. In some models, WTP is transformed using logarithms because it is right-skewed, as ln(WTP) better captures diminishing marginal willingness. Additionally, the models incorporate indicators for whether the participant is a beginning farmer and self-assessments of knowledge in more comprehensive models. Equations estimated for the five models presented in Table 2 are as follows:

**Table 2:** Results from simple linear model of estimating willingness to accept (*WTA*).

Model #	(2.1)	(2.2)	(2.3)	(2.4)	(2.5)
Explanatory Variables	*WTA*	*WTA*	*WTA*	*WTA*	*WTA*
*WTP*	0.795*				
	(0.414)				
*Ln(WTP)*		69.92**	65.10**	64.27**	64.74**
		(28.85)	(30.86)	(30.89)	(30.22)
*TravC*			−0.0545		
			(0.309)		
*TotC*				−0.106	
				(0.290)	
*DBegFarm*					100.5*
					(50.57)
*AvgKnowBef*					35.88
					(28.95)
*AvgKnowAft*					−75.20**
					(35.77)
*Constant*	254.8***	35.40	50.66	71.36	204.5
	(35.13)	(114.8)	(125.0)	(130.5)	(150.2)
Observations	67	67	64	64	63
Shapiro-Wilk (Prob)	0.000	0.000	0.000	0.000	0.008
Adj R-squared	0.034	0.064	0.035	0.037	0.136
AIC	892.2	890.1	851.7	851.6	833.1
BIC	896.6	894.5	858.2	858.1	843.9
Log Likelihood	−444.1	−443.1	−422.9	−422.8	−411.6
Root MSE	185.8	182.9	183.5	183.4	173.3
Statistics using the total number of common observations (61) for all models					
Adj R-squared	0.033	0.064	0.054	0.06	0.168
AIC	810.2	808.2	809.8	809.4	803.8
BIC	814.4	812.4	816.1	815.7	814.4
Log Likelihood	−403.1	−402.1	−401.9	−401.7	−396.9
Root MSE	182.3	179.4	180.3	179.8	169.1

Note: Robust standard errors are in parentheses. *,** and *** indicate 10%, 5%, and 1% statistical significance levels, respectively.


$$\begin{array}{c} WTA_{i}=\alpha_{\text{1}}+\alpha_{11}WTP_{i}+\varepsilon_{1i} \end{array}$$ (2)

$$\begin{array}{c} WTA_{i}=\alpha_{\text{2}}+\alpha_{21}Ln\left(WTP_{i}\right)+\varepsilon_{2i} \end{array}$$ (3)

$$\begin{array}{c} WTA_{i}=\alpha_{\text{3}}+\alpha_{31}Ln\left(WTP_{i}\right)+\alpha_{32}TravC_{i}+\varepsilon_{3i} \end{array}$$ (4)

$$\begin{array}{c} WTA_{i}=\alpha_{\text{4}}+\alpha_{41}Ln\left(WTP_{i}\right)+\alpha_{42}TotC_{i}+\varepsilon_{4i} \end{array}$$ (5)

$$\begin{array}{c} WTA_{i}=\alpha_{\text{5}}+\alpha_{51}Ln\left(WTP_{i}\right)+\alpha_{52}DBegFarm_{i}+\alpha_{53}AvgKnowBef_{i}+\alpha_{54}AvgKnowAft_{i}+\varepsilon_{5i} \end{array}$$ (6)

where *WTA*_*i*_ is the lowest dollar value presented that participant *i* is willing to accept to leave with no educational materials before the scheduled in-person training begins; *WTP*_*i*_ is the dollar amount that participant *i* reports they are willing to pay for the training before the scheduled education begins; *Ln(WTP)*represents the logarithm of *WTP*. *TravC* is the estimated transportation and travel time cost associated with individual *i* attending the training; *TotC* (*TravC + cost of attendance (OPCattend*)) is the estimated total cost associated with individual *i* attending the training by adding their opportunity cost to attend the training to *TravC*; and $\varepsilon_{1i}$, …, $\varepsilon_{5i}$ denote the *i*^th^ residual for their respective equations in (2) through (6) corresponding to the respective models in (2.1) through (2.5) in Table 2. After testing the error structure using the Shapiro-Wilk test for normality [32], we also tested for the presence of heteroskedasticity using the White test [30,33]. Results of these different tests are presented in Table 2. While these regressions reveal basic correlations, the Shapiro–Wilk test for residual normality (shown at the bottom of Table 2) consistently rejected normality (p-values = 0.000).

#### 4.2.2. Ordered probit specification.

Considering that participants’ WTA selections were categorical and naturally ordered ($25 < $50 < $71.5 < $100 < $125 < $250 < $500), an ordered Probit model was employed. Unlike binary logit or probit, which compresses ordinal responses into a yes or no format, or multinomial logit which treats outcomes as nominal rather than ranked, ordered Probit uses threshold cutoff points that preserve the ordinal nature of WTA.

Model fit statistics of Akaike Information Criterion (AIC), Bayesian Information Criterion (BIC), and log-likelihood in Tables 2 and 3 demonstrate that the ordered Probit framework more effectively captures the distribution of WTA outcomes than OLS, as indicated by lower AIC and BIC values. In contrast, the OLS residuals consistently failed normality tests, further supporting that a linear model is inappropriate for the ordinal response data. Therefore, we utilized an ordered Probit framework for formally analyzing these data.

**Table 3:** Results of ordered probit regression estimation of willingness to accept (*WTA*) as a function of willingness to pay *(WTP)* and other explanatory variables as described in Table 1.

Model #	(3.1)	(3.2)	(3.3)	(3.4)	(3.5)
Explanatory Variables	*WTA*	*WTA*	*WTA*	*WTA*	*WTA*
*Ln(WTP)*	0.490***	0.517**	0.491**	0.503**	0.463**
	(0.186)	(0.226)	(0.236)	(0.232)	(0.234)
*DBegFarm*		0.728**	0.832**	0.739*	0.730*
		(0.324)	(0.339)	(0.410)	(0.416)
*AvgKnowBef*		0.367*	0.425**	0.372*	0.398*
		(0.206)	(0.215)	(0.207)	(0.210)
*AvgKnowAft*		−0.663**	−0.835***	−0.692**	−0.783***
		(0.279)	(0.296)	(0.279)	(0.296)
*TravC*			0.0004		0.0002
			(0.002)		(0.002)
*Education*				−0.038	−0.025
				(0.075)	(0.076)
*Male*				0.233	0.210
				(0.300)	(0.303)
*Age*				−0.003	−0.004
				(0.011)	(0.011)
Observation	67	63	61	62	61
Shapiro-Wilk (Prob)^+^	0.748	0.959	0.795	0.851	0.787
Pseudo R-squared	0.034	0.090	0.106	0.112	0.111
AIC	216.3	195.2	186.9	195.6	192.0
BIC	231.7	216.7	210.1	223.2	221.5
Log Likelihood	−101.1	−87.61	−82.44	−84.79	−81.98
Statistics using the total number of common observations (61) for all models					
Pseudo R-squared	0.035	0.106	0.106	0.111	0.111
AIC	192.1	184.9	186.9	190.0	192.0
BIC	206.9	206.0	210.1	217.4	221.5
Log Likelihood	−89.05	−82.46	−82.44	−81.99	−81.98

Note: Robust standard errors are in parentheses. *,** and *** indicate 10%, 5%, and 1% statistical significance levels, respectively. ^+^ Not a residual normality test, just distribution of the linear predictor

Because other factors are also likely to influence a participant’s WTA bid, we include additional variables such as farming or ranching experience and education. We present five different models to demonstrate the robustness of our variables considered in estimating participants’ WTA values (Table 3). The equation corresponding to model (3.5) estimated in Table 3 is as follows:


$$\begin{array}{c} \begin{array}{c} WTA_{i}^{*}=\beta_{51}Ln\left(WTP_{i}\right)+\beta_{52}TotC_{i}+\beta_{53}DBegFarm_{i}+\beta_{54}AvgKnowBef_{i}+\beta_{55}AvgKnowAft_{i}\mathrm{\backslash hfill}\\ +\beta_{56}\begin{array}{c} Education_{i} \end{array}+\beta_{57}Male_{i}+\beta_{58}\begin{array}{c} Age_{i} \end{array}+\varepsilon_{i}\mathrm{\backslash hfill} \end{array} \end{array}$$ (7)

Where $WTA_{i}^{*}$ represents the latent variable for WTA; *DBegFarm*_*i*_ *is a* dummy variable for beginning farmer (*DBegFarm* = 0 if farming or ranching for more than 10 years; else = 1); *AvgKnowBef*_*i*_ represents the average level of self-assessed prior knowledge by respondent *i* on topics addressed; *AvgKnowAft*_*i*_ similarly represents knowledge on the same topics at the end of the training; *Education*_*i*_ represents the number of school years achieved by respondent *i*. *Male*_*i*_ represents a dummy variable when the gender of respondent *i* is male (*Male* = 1 and 0 if female), *Age*_*i*_ represents the age of respondent *i* and $\varepsilon_{i}$ are error terms with the assumption that they follow a standard normal distribution, and other variables are as defined earlier.

#### 4.2.3. Robustness checks.

We conducted multiple robustness checks to confirm the reliability of our findings. First, we used robust (heteroskedasticity-consistent) standard errors [30] for all regressions, both OLS and ordered probit. This method addresses concerns related to heteroskedasticity, which arises from varied sample characteristics. For instance, some participants may show notably greater WTA variance. Consistent conclusions are that WTP is significant in both OLS and ordered probit models, and knowledge variables are significant in the ordered probit regardless of whether we applied conventional or robust standard errors. This suggests that although heteroskedasticity is present, it does not distort our primary results. We also examined different specifications for travel costs. For example, we replaced total cost (*TotC)* with travel costs (*TravC)* in the ordered probit, which aligns with OLS Model 2.4, resulting in a small and statistically insignificant coefficient. Moreover, we computed measures, such as WTA minus travel cost and WTP plus travel cost (definitions available in Table 1), to evaluate if WTA reflected the need to cover travel expenses. Nevertheless, regression models using these combined travel cost metrics did not yield any statistically significant results. We also explored whether a respondent traveling alone or with others would have an influence on their WTA choice, but the results were not statistically significant. The impact of WTP remained robust on explaining WTA values, and travel cost measures were not statistically significant for explaining WTA. Restricting the OLS and ordered-probit models to the common estimation sample (N = 61) so the two are directly comparable, the ordered-probit specification continued to fit better than OLS (lowest common-sample AIC and BIC for model 3.2), further supporting our model choice. Finally, although our primary categorical model is probit, we conducted a robustness check with an ordered logit, which yielded coefficients with consistent signs and similar significance levels across all covariates, confirming the stability of our results regardless of the function selected.

Because perceived program benefits could also plausibly influence the compensation participants require to forgo a training session, we re-estimated the full specification (Models 2.5 and 3.5) using the common regression subsample of 58 participants, adding *PerceivedBenefit* (equal to one if the participant believed the session would ‘definitely’ improve their success versus ‘probably’ or ‘maybe’) and *SolidifiedCommit* (equal to one if the participant reported that the session solidified their commitment to farming or ranching). The signs and magnitudes of the coefficients on models 2.5 and 3.5 remained substantively stable. The beginning farmer or rancher indicator remained positive and marginally significant across the robustness models (p = 0.079–0.087), consistent with the main results. Neither added measure was statistically significant (*PerceivedBenefit*: OLS p = 0.76, ordered probit p = 0.82; *SolidifiedCommit*: OLS p = 0.53, ordered probit p = 0.49), and post-training self-assessed knowledge continued to be negative and statistically significant (ordered probit p = 0.008; OLS p = 0.024). *Ln(WTP)* stayed positive and of similar magnitude (ordered probit changing from 0.490 to 0.467 and OLS from 67.8 to 65.0) but became marginally significant, just above the 5% threshold, in the augmented specifications (ordered probit p = 0.060; OLS p = 0.067); in the base models on this subsample, it was significant at the 5% level in the ordered probit (p = 0.043) and at the margin in OLS (p = 0.050). Collinearity diagnostics did not indicate a concern. The mean of the variance inflation factor (VIF) was 1.48, the maximum VIF was 2.03, and the VIFs for the two added measures were 1.72 and 1.20. The added measures were only modestly correlated with knowledge variables (e.g., *PerceivedBenefit* with post-training knowledge, r = 0.23). Full results are shown in S1 Table.

For model diagnostics, we utilized the Shapiro–Wilk test on the distribution of linear predictors of the ordered probit models and found no significant deviation from normality (p > 0.1 in most instances), unlike the OLS residuals. This finding supports the probit model’s assumption of a normally distributed latent propensity for selecting higher WTA. In summary, these robustness checks confirm that the estimated relationships are substantial and not just products of specific model assumptions or sample subsets.

#### 4.2.4. Marginal effects.

For an ordered probit model, the coefficients β shift the latent variable $y_{i}^{*}$ [34,35], but the effect on each outcome probability is nonlinear. Thus, we use marginal effects to measure how a small change in *x*_*k*_ affects P(*y*_*i*_ *= j*). Specifically, the marginal effect of a continuous variable *x*_*k*_ on the probability that *y*_*i*_ *= j* is:


$$\begin{array}{c} \frac{\partial }{\partial x_{k}}P(y_{i}=j|X_{i})=\left[\phi (\alpha_{j}-{X^{\prime }}_{i}\beta )-\phi (\alpha_{j-1}-{X^{\prime }}_{i}\beta )\right]\times \beta_{k}, \end{array}$$ (8)

where ϕ(·) denotes the standard normal probability density function (pdf), $\frac{\partial }{\partial x_{k}}\left({X^{\prime }}_{i}\beta \right)=\beta_{k}$, $\phi (\alpha_{j}-{X^{\prime }}_{i}\beta )$ represents the pdf evaluated at the “upper threshold” for category *j* and $\phi (\alpha_{j-1}-{X^{\prime }}_{i}\beta )$ denotes the pdf at the “lower threshold”. Their difference scales by **β**_k_ to give the net change in probability for category *j* due to a small increase in *x*_*k*_. For *j* = 1, the lowest category, then since $\alpha_{\text{0}}=-\infty ,\text{\textbackslash{}hspace\{0.17em}\;\;\;\;\;\;\}\phi (\alpha_{\text{0}}-x^{\prime }\beta )=\phi (-\infty )=0$, or the lower limit. Thus,


$$\begin{array}{c} \frac{\partial }{\partial x_{k}}P(y=1)=-\beta_{k}\phi (\alpha_{\text{0}}-x^{\prime }\beta ). \end{array}$$ (9)

If $\beta_{k}$ >0, this derivative is negative, meaning an increase in *x*_*k*_ reduces the probability of being in category 1. For *j* = *J* (the highest category), $\alpha_{J}=+\infty$, so $\phi (\alpha_{J}-x^{\prime }\beta )=0$, yielding

$\frac{\partial }{\partial x_{k}}P(y=1)=-\beta_{k}\phi (\alpha_{\text{0}}-x^{\prime }\beta )$. Hence for $\beta_{k}>0$, an increase in *x*_*k*_ *raises* the probability of the highest category.

As an example**,** suppose in Model 3.2 (Table 3) $\beta_{\text{1}}>0$ is the coefficient for *Ln(WTP)*. Then, from Equation (8) for each WTA category *j*,


$$\begin{array}{c} \frac{\partial }{\partial Ln\left(WTP\right)}P(y=j)=\left[\phi (\alpha_{j}-x^{\prime }\beta )-\phi (\alpha_{j-1}-x^{\prime }\beta )\right]\times \beta_{Ln\left(WTP\right)}. \end{array}$$ (10)

Evaluating at the sample means, one can compute the numerical effect. For instance, if at the means we have $(\alpha_{\text{6}}-x^{\prime }\hat{\beta })\approx -0.068$ and $\beta_{Ln\left(WTP\right)}=0.517$, then ϕ(−0.068)≈0.3985, so $\frac{\partial }{\partial Ln\left(WTP\right)}P(y=7)\approx 0.3985\times 0.517\approx 0.206$. This matches the STATA output of 0.204 from Table 4. This implies a one-unit rise in *Ln(WTP)* (roughly doubling *WTP*) raises the probability of being in the highest *WTA* category by about 26 percentage points, everything else held constant.

**Table 4:** Marginal effects at different WTA levels for ordered probit models using models (3.1) and (3.2).

	Model (3.1) (N = 67)	Model (3.2) (N = 63)			
Marginal effect (at means) given	*Ln(WTP)*	*Ln(WTP)*	*DBegFarm*	*AvgKnowBef*	*AvgKnowAft*
WTA = 25	−0.025	−0.016	−0.029	−0.011	0.020
	(0.019)	(0.012)	(0.022)	(0.010)	(0.016)
WTA = 50	−0.056*	−0.047	−0.074	−0.033	0.060
	(0.029)	(0.029)	(0.045)	(0.028)	(0.041)
WTA = 71.5	−0.034*	−0.032	−0.046	−0.022	0.041
	(0.020)	(0.022)	(0.034)	(0.014)	(0.025)
WTA = 100	−0.053**	−0.068*	−0.090*	−0.048	0.088*
	(0.026)	(0.038)	(0.052)	(0.032)	(0.048)
WTA = 125	−0.011	−0.018	−0.021	−0.013	0.023
	(0.008)	(0.013)	(0.014)	(0.009)	(0.016)
WTA = 250	−0.015	−0.025	−0.016	−0.017	0.031
	(0.016)	(0.023)	(0.027)	(0.016)	(0.027)
WTA = 500	0.193***	0.204**	0.277**	0.145*	−0.262**
	(0.073)	(0.090)	(0.113)	(0.081)	(0.110)

Note: Robust standard errors are in parentheses. *, ** and *** indicate 10%, 5%, and 1% statistical significance levels, respectively.

If $x_{k}$ is a dummy (binary) variable like when *DBegFarm* = 0 vs. 1, the marginal effect is computed as: $\Delta_{j}=P(y=j|x_{k}=1)-P(y=j|x_{k}=0)$ while holding other variables at reference levels. That is, we do not take a derivative but compare predicted probabilities under each scenario. The difference operator, $\Delta_{j}$, indicates how the dummy variable shifts the probability of each outcome.

## 5. Results

Participants’ WTA to forgo training sessions covered all seven discrete WTA offers with a mean WTA of $277.44 (median of $250). In contrast, their WTP averaged only $58.47 (median $50). As expected, WTA values are much larger than the WTP values of participants. The mean and median WTA values are $69.36 and $62.50 per hour of education, considering four-hours of training per session. Whereas, the corresponding WTP values are only $14.62 and $12.50 per hour of education.

Participants incurred costs to attend as they traveled an average of 88.9 miles one-way (median 55 miles) and they often traveled with others (mean travel party size of 1.86 people), as shown in Table 1. Estimated travel costs averaged $69.08, and combined with the opportunity cost of time on-site, the total cost of attending was about $110 on average. Despite these costs, participants perceived their benefits to outweigh these costs. Indeed, the average net benefit (WTA minus travel costs) value is roughly $209, and shows that the average WTA far exceeds travel costs. It’s also informative to note that adding travel costs to WTP (i.e., total willingness to incur cost) yields a mean of $131, still well below the mean WTA of $277.44, underscoring the difference in these value measures.

*WTP* values were generally low relative to travel cost (TravC), with mean WTP ($58.47) below mean TravC ($69.08), although median WTP ($50) slightly exceeded median *TravC* ($42.25). Mean and median TravC were $69.08 and $42.25 for the event, or $17.27 and $10.56 per hour of in-person education. On average, *WTA* values are 4.74 times larger than *WTP* without adjusting for *TravC* and 2.11 times larger when adjusted for *TravC (WTP + TravC)*. This large gap is consistent with the well-documented disparity between WTA and WTP for the same good. Such differences between WTA and WTP have been observed in many valuation studies [3,4](Hanemann 1991; Horowitz & McConnell 2002). These ratios are close to the *WTA/WTP* ratios of 2.35 to 3.56 found by Frondel et al. [11] for security of electrical power supply but noticeably lower than the more than seven-fold average ratio observed by Horowitz and McConnell [3].

Our sample consists of a mix of beginning farmers and ranchers and more experienced producers. 66% of respondents identified themselves as beginning farmers or ranchers (≤10 years in farming/ranching), our target audience. On average, for four hours of in-person training, beginning farmers and ranchers report a higher WTA of $305.07 compared to $223.89 for their more experienced counterparts, 36.3 percent more. Similarly, the average WTP among beginning farmers or ranchers is $66.88, 51.5 percent more than the $44.15 average reported by experienced farmers. They require an additional $20.30 per hour of education to opt out the training, and they are willing to pay $5.68 more per hour of training to attend.

Our sample of participants is roughly half male (52%), and the age categories selected range widely, with an average of 48.5 years (age categories of <20, 20–24, 25–34, 35–44, 45–54, 55–64, and ≥65 years). Education levels were fairly high with a mean education of 14.9 years and median of 14 (education categories of High School (12), Some Technical (14), Associates Degree (14), Bachelors Degree (16), and Graduate Degree (20)). One aspect of each training’s impact is reflected in their self-assessed knowledge scores. Participants rated their knowledge of the topics before attending the training as only a 2.20 average on a 5-point likert scale (1 poor, 2 fair, 3 good, 4 very good, and 5 excellent). Self-assessed knowledge ratings rose to 3.67 at the end of the training. Since participants came and left with different self-assessed knowledge levels, we utilize these as independent variables for explaining *WTA* responses. Distributions of two additional collected perception variables, perceived benefit of the session and its influence on participants’ future direction, are provided in S2 Table; these variables are included in the sensitivity check in Section IV.2.3.

When analyzing participants’ logistics, we found that 43.36% traveled alone, while the remaining 56.64% traveled with at least one other person. Specifically, 33.63% traveled with one companion, 16.81% with two companions, and 6.19% with three or more. The questionnaire recorded the number of travel companions but not their relationship to the participant, so we cannot determine whether companions were family members, friends, or unrelated community members; we therefore do not infer the composition of travel parties. Regarding travel expenses, the overwhelming majority of respondents (84.21%) covered their own transportation costs, while only 11.40% had their expenses reimbursed by an employer or organization, and 4.39% received assistance from family members.

The ordered probit estimates in Table 3 reveal a consistent, highly significant positive relationship between *Ln(WTP)* and *WTA* across all model specifications. The coefficient increases from 0.49 in the baseline model (3.1) and ranges from about 0.46 to 0.52 across the augmented models, remaining significant at least at the 5 percent level even after additional controls in model (3.5). This indicates that participants who report a greater *WTP* also seek significantly higher compensation to forgo the training. Beginning farmers and ranchers (*DBegFarm*) show coefficients ranging from 0.73 to 0.83, significant at the 5–10 percent levels in models (3.2) through (3.5), confirming that new farmers value training more than their experienced counterparts; the effect remains marginally significant (p < 0.10) even in the fully specified model. Self-assessed knowledge before the session (*AvgKnowBef*) has a positive correlation with WTA, with estimates ranging from approximately 0.37 to 0.43 at the 5% or 10% level, while knowledge gains post-session (*AvgKnowAft*) negatively correlate, ranging from –0.66 to –0.84 at either the 5% or 1% level. This suggests that once participants feel they have mastered the subject at a high level, the compensation they would require to not attend a training session decreases. No statistically significant influence is found for travel costs, education, gender or age, indicating that these factors do not affect WTA when considering WTP and knowledge measures. Model fit improves with additional controls, as evidenced by the pseudo-R² increasing from 0.034 to 0.111 across models 3.1 to 3.5; on the common estimation sample (N = 61), model (3.2) has the lowest AIC (184.9) and BIC (206.0), indicating that the model specification without travel cost, education, gender and age (model 3.2) best reflects the factors influencing participants’ compensation expectations of our models.

While the ordered probit coefficients tell us the direction of influence on the latent WTA, marginal effects are calculated to show the impact of these variables on the probabilities of each WTA outcome. Table 4 reports the marginal effects of selected variables on the probability of choosing each of the seven WTA amounts, evaluated at the sample means of other covariates. Model 3.2 has the lowest AIC and BIC values of the five models shown in Table 3 using the same observations. Thus, Table 4 shows results for two models: Model 3.1 (*Ln(WTP)*) and Model 3.2 (*Ln(WTP)*+ *DBegFarm* + *AvgKnowBef* + *AvgKnowAft*), to illustrate how additional factors shift the distribution of WTA. We summarize key insights from these marginal effects, focusing on Model 3.2, which includes the main covariates of interest and has the lowest comparable AIC and BIC values.

An increase in *Ln(WTP)* significantly shifts the probability mass from lower to higher *WTA* amounts. For example, holding other variables at their means, a one-unit increase in *Ln(WTP)* (approximately a 171% increase in actual *WTP*) reduces the probability of accepting $50 by about 5.6 percentage points (p < 0.10), and reduces the probability of $100 *WTA* by 5.3 points (p < 0.05). Concurrently, it increases the probability of requiring $500 (the highest category) by about 19.3 points (p < 0.01). In other words, a participant with a higher underlying WTP is much less likely to accept a smaller compensation (i.e., ≤ $100) to leave and much more likely to accept only the maximum $500. These marginal effects underscore our robust result that WTP is a strong driver for explaining WTA. That is, those who value a training session more as expressed by a high WTP are disproportionately represented among those who will only leave for a very high WTA payoff. Conversely, someone with a low WTP has a higher chance of accepting a lower compensation to skip the training. The monotonic pattern for *Ln(WTP)* with negative marginal effects at all the low/mid WTA levels and a large positive effect at the highest level reflects an upward shift of the entire WTA distribution as WTP increases.

Being a beginning farmer or rancher also shifts the probability upwards to higher WTA categories, though the effects are most pronounced at the very top category. A beginning farmer or rancher is slightly less likely to accept lower offers (for instance, the probability of $50 WTA is 7.4 percentage points lower for a beginning farmer or rancher, though not significant at 10% in Model 3.2), and notably more likely to demand $500. Specifically, the marginal effect for beginning farmers and ranchers on the $500 category is 27.7 percentage points (p < 0.05). This indicates that, all else equal, a beginning farmer or rancher has roughly a 28 percent greater chance of being in the highest WTA group compared to a more experienced farmer. This is consistent with our earlier coefficient interpretation of new farmers being more inclined to require the maximum compensation to skip the training. While many of the marginal effects for intermediate categories are not individually significant for beginning farmers and ranchers, the overall pattern suggests that being a beginner leans one toward the higher end of WTA.

Self-assessed knowledge before the training (*AvgKnowBef*) shows a similar directional influence as being a beginning farmer or rancher. The positive statistically significant coefficient increases the likelihood of higher WTA levels at the highest category of $500. For instance, when the knowledge-before rating increases by one unit (e.g., from “Fair” to “Average”), there is a corresponding 4.8 percentage point drop in the probability of a $100 WTA (though not statistically significant). Conversely, at the upper extreme, this same rise in prior knowledge increases the probability of needing $500 by approximately 14.5 points (p < 0.10). Therefore, individuals who report as more knowledgeable before the training begins are more likely to value the education proposed, especially at the highest WTA payout. That is, those who have a higher self-assessed knowledge before a training session are less likely to forgo the class without a substantial reward. The most pronounced impact occurs at the highest level (+14.5%), while a balancing decrease is observed across the lower tiers, although these effects are not statistically significant.

The marginal effects of self-assessed post-training knowledge are essentially a mirror-image of prior knowledge. A higher self-assessed knowledge after the training (which was associated with a lower latent WTA) increases the probability of lower WTA categories and decreases the probability of the highest category. In Model 3.2, a one-unit increase in self-assessed knowledge-after significantly increases the probability of accepting $100 by 8.8 points (p < 0.10) and reduces the probability of $500 by 26.2 points (p < 0.05). We also see positive (though not significant) marginal effects in some of the lowest categories (e.g., + 2.0% for $25 and +6.0% for $50). Individuals who self-assessed as very knowledgeable after the training, whether from information obtained prior to the training or during the training, are more likely to be in a lower WTA bracket initially. For example, someone whose post knowledge is “Excellent (5)” vs “Good (4)” has about a 26 percentage point lower chance of requiring $500 to skip the training. A higher level of before-knowledge may mean that these individuals better understand what they can actually benefit from the training while higher after-knowledge indicates that these individuals have possibly already mastered the education presented. Beginning farmers and ranchers were the most insistent on a high payoff to leave, reinforcing our earlier interpretation that they likely have the most to benefit from the training sessions.

## 6. Conclusions

We constructed and implemented a new experimental methodology to quantify the value of education using WTA, particularly for limited resource audiences. By allowing participants to “cash out” of a training session, we observed the minimum value they are willing to forgo a training session instead of receiving the education scheduled. This experimental design is constructed to elicit incentive-compatible responses and mitigate the income-bias limitations of standard WTP surveys by employing WTA as the metric. In training sessions for beginning farmers or ranchers, we found that participants value the training at approximately $69.36 per hour, a figure that significantly surpasses their stated WTP of $14.62 per hour. This indicates that conventional evaluation methods, such as surveys asking for WTP or relying on nominal fees, may greatly underestimate the actual value of educational programs, especially for low-income individuals. To account for the ordinal structure of our WTA responses, we estimate ordered probit models and find that *Ln(WTP)* significantly predicts most WTA levels, especially at the high end (WTA = $500). A one-unit increase in *Ln(WTP)* raises the probability of selecting the highest WTA ($500) by 19.3 to 20.4 percentage points, while decreasing the chances of selecting the mid-to-lower WTA $100 category by roughly 5–7 percentage points. Our findings are consistent with the broader literature on WTA versus WTP, which shows that individuals require much greater compensation to forgo a good than they are willing to pay for it. Notably, we found that the intended beneficiaries, new and limited-resource farmers, are the ones who place the highest dollar value on the training sessions. Beginning farmers and ranchers are more likely to demand higher WTA amounts, especially the maximum of $500. They show a 27.7 percentage point increase in selection probability for $500, while higher beginning prior knowledge increases the likelihood of selecting $500 by 14.5 points. This substantiates the case for publicly funding the education of individuals with limited resources, as for them to cover the full cost would likely prevent many from participating, even though their non-income-constrained benefits identified through this experiment are considerably more than their estimated total travel costs and opportunity cost of time for attending.

Our estimated WTA/WTP ratios of 2.11 to 4.74 fall within the range documented in the broader literature, below the roughly seven-to-one ratio reported by Horowitz and McConnell (2002) [3] and the above-six ratio for environmental goods in Tunçel and Hammitt’s (2014) [10] meta-analysis, near their three-to-one average across goods, and close to the 2.35–3.56 ratio reported by Frondel et al. (2021) [11]. That our gap is moderate rather than extreme is consistent with the meta-analytic finding that incentive-compatible elicitation with real stakes narrows the disparity relative to hypothetical methods, supporting the value of our field design. Our results also point to a channel beyond the endowment effect emphasized by Kahneman, Knetsch, and Thaler (1990) [5]: for limited-resource participants the WTA-WTP gap partly reflects a binding income constraint on WTP [4], not loss aversion alone. Substantively, our finding that beginning farmers and ranchers report the highest WTA complements the extension-valuation literature [17,18,21], which values extension chiefly through income-constrained WTP and may therefore understate its worth to those with the most to gain.

Recent valuation evidence on extension comes largely from developing-country digital-advisory settings, where incentive-compatible and stated-preference elicitation finds WTP that is strongly conditioned by ability to pay and often near or below the cost of provision [17,18,21]. Our results extend this evidence in two directions: to in-person education in a high-income country, and from payment-based to compensation-based valuation, which is not mechanically capped by participants’ budgets. Methodologically, by adapting the incentive-compatible logic of BDM and experimental auctions [14–16] to a field education setting, our approach offers program evaluators a pragmatic design for providing a demand-revealing alternative to hypothetical WTP for limited resource audiences.

For educators and program evaluators, the WTA approach offers a valuable framework for quantifying the financial worth of educational outcomes. The derived dollar amounts can assist with cost-benefit analyses, grant reporting, and more accurately communicating the financial impacts of programs. Our research also emphasizes the importance of considering participants’ WTP and existing knowledge when interpreting valuation data; acknowledging the gap between WTA and WTP can help clarify why participants may report they won’t pay much to attend a training session, yet they still hold the training in high esteem, as shown by WTA.

Participants indicating a higher WTA are beginner farmers and those having a higher before-knowledge. In contrast, individuals with high post-training knowledge (either knowledge obtained prior to the training or at the training) require low to mid WTA outcomes with the highest WTA being the least likely. These effects were mostly statistically significant at conventional levels for the extreme WTA values. Probabilities of intermediate WTA amounts (e.g., $71.5, $125, $250) generally adjust in opposite directions to balance out the changes. Many of those shifts are small or not individually significant, which is expected since the strongest movements tend to be at the distribution tails for an ordered model.

Our instrument did not include validated measures of institutional trust or of risk and time preferences, which the valuation literature identifies as potential determinants of stated value [19,20]. As a partial check, we added the two perception items (perceived benefit of the session and its influence on the participant’s future direction) to the fully specified models; both were statistically insignificant and did not change the sign or interpretation of the main covariates, and *Ln(WTP)* remained positive and similar in magnitude but became marginally significant, falling just above the 5% threshold in these augmented specifications, possibly reflecting the limited sample size and overlap between stated valuation and perception measures. We therefore cannot rule out that trust in the sponsoring institution or individual risk attitudes contribute to the WTA-WTP gap we observe. We flag the direct measurement of these factors, for example, with established trust and risk-preference instruments [20], as a priority for future field valuations of education.

In conclusion, the WTA experimental method presented shows promising potential for valuing educational services, especially for participants with limited resources. It shows that participants derive significant economic value even when they are unwilling or cannot afford to pay a substantial fee. This insight is essential for justifying and improving resource allocation for educational programs designed for those who arguably need them the most. Future research could apply this methodology to different educational settings and audiences to better quantify how individuals value learning opportunities. Overall, our analysis and results indicate that soliciting WTP is preferred to utilizing detailed travel cost estimates for obtaining an individual’s WTA cash payout for education.

## Supporting information

S1 Appendix Participant questionnaire/evaluation instrument (blank). (PDF)

S1 Table Robustness check: perceived-benefit variables added to the fully specified OLS and ordered-probit models (Models 2.5 and 3.5). (PDF)

S2 Table Distributions of collected perception items included in the public de-identified dataset. (PDF)
