# Supplementary material for: Valuing education for limited-resource audiences using a willingness to accept experiment
Source: PLoS One. 2026 Sep 16;21(9):e0348858. doi: 10.1371/journal.pone.0348858 (PMC13581028; doi:10.1371/journal.pone.0348858)
Supplement: Supplementary file 1 [file pone.0348858.s001.pdf]

S1 Appendix. Participant questionnaire/evaluation instrument (blank)

Blank reconstruction of the two closely related instruments used at the two in-person training sites. No respondent answers are included. The two forms shared a common set of items and a common willingness-to-accept / willingness-to-pay (WTA/WTP) elicitation, and differed only in a small number of site-specific items and in their knowledge-assessment topics. Bracketed terms (e.g., "[farming/ranching]") indicate site-specific wording. Response options are shown blank.

Module 1: Common questions (administered at both sites)

1. How far did you travel (one-way, in miles) to attend today's [class/training]? ______ miles (one-way)

2. Did you come by yourself or travel with others? *(select one)* ☐ Self ☐ 1 other ☐ 2 other ☐ ≥ 3 other

3. How do you classify yourself? *(select one)* ☐ Interested in [farming/ranching] someday ☐ Been [farming/ranching] less than 10 years ☐ Been [farming/ranching] more than 10 years ☐ Other: ______

4. How would you classify your [crop-growing / ranching] experience in Arizona? *(select one)*

- Small-town / beginning-farmer site: ☐ Expert (> 5 harvests) ☐ Experienced (2-5 harvests) ☐ Novice (1-2 harvests) ☐ Planted but no harvest ☐ No experience
- Rural-area / beginning-rancher site: ☐ Expert (> 10 calf crops) ☐ Experienced (5-10 calf crops) ☐ Moderate (2-5 calf crops) ☐ Novice (1-2 calf crops) ☐ No experience

5. What is the highest formal education you have achieved? *(select one)* ☐ High School ☐ Some Technical College ☐ Associate's Degree ☐ Bachelor's Degree ☐ Graduate Degree

6. What is your age? *(select one)* ☐ < 20 ☐ 20-24 ☐ 25-34 ☐ 35-44 ☐ 45-54 ☐ 55-64 ☐ ≥ 65 years

7. What is your race/ethnicity? *(select one)* ☐ Native American ☐ African-American ☐ Hispanic ☐ Non-Hispanic White ☐ Asian ☐ Multi-racial ☐ Other

8. What is your gender? *(select one)* ☐ Male ☐ Female ☐ Other: ______

9. How many UA Cooperative Extension programs (including the classes in this series) have you attended in the last five years? *(select one)* ☐ None ☐ 1-3 ☐ 3-5 ☐ 5-10 ☐ > 10

10. If you have attended any UA Cooperative Extension programs before, have you implemented any new management practice you learned? *(select one)* ☐ Yes ☐ No. If yes, which practices? ______

11. Do you believe today's [classes/training] would or will improve your success? *(select one)* ☐ Definitely ☐ Probably ☐ Maybe ☐ No Difference

12. How have today's [classes/training] influenced your direction regarding being a future [farmer/rancher]? *(select one)* ☐ Solidified current commitment to [farming/ranching] ☐ Definitely like to start some day ☐ No change / Undecided ☐ Convinced me not to [farm/ranch]

13. What suggestions do you have to make the [classes/training] better? ______

Module 2: Willingness-to-Accept / Willingness-to-Pay elicitation (common to both sites; voluntary)

In this section, please circle your "Willingness-To-Accept" to leave before the training begins in A and write down your "Willingness-To-Pay" for today's training in B.

A: If you were asked to leave today's training without receiving any educational presentations or materials, what is the lowest amount you are Willing-To-Accept to leave before any training begins?

(Please notice, if you are the one selected from the drawing, you will receive the amount you choose but you will be required to leave the workshop immediately with the compensation. Your selection of one of the 7 amounts below will not be revealed to anyone in today's training.)

There are 2 parts for the drawing:

(1) There are 49 tickets in the first drawing. 20 tickets with $25, 10 with $50, 7 with $71.5, 5 with $100, 4 with $125, 2 with $250, and 1 ticket with $500. A single compensation amount will be randomly drawn among the 49 tickets.

(2) After the first drawing, if say $100 is the compensation amount drawn, we will then perform another drawing among all those who selected $100 as their "Willingness-To-Accept." If you are the one selected under this second part, we will provide you the compensation you selected, but you will be asked to leave immediately.

In general, higher Willingness-To-Accept values are less likely to be drawn and less likely to require you to leave before the training begins. Please note that "A" and "B" in this box are voluntary and that your participation will help quantify the monetary value of trainings like this. Please participate by identifying the lowest $ amount below that you would be willing to accept in exchange for leaving today's class immediately.

Part A.	***Please circle the lowest dollar amount in compensation below that you would be willing to accept in order to forego attending today's training AND write this amount on the ticket to hand in (circle only one $ amount).***						
Dollars in compensation you will accept to leave today's training. (circle/write only one $ amount).	**$25**	**$50**	**$71.5**	**$100**	**$125**	**$250**	**$500**
	(write the same $ amount on your ticket that is circled in row above)						
**# of Tickets (49 total) for each $ Amount in row above**	**20**	**10**	**7**	**5**	**4**	**2**	**1**

If your ticket is drawn, please realize that you will receive the monetary compensation you selected ***AND*** *will also need to* ***leave without receiving the educational presentations and materials of today's training****. No one will know how much you "bid" for your compensation needed to leave today's class as the dollar amounts will not be made public. The expected payout for $25 (20/49*$25=$10.20) equals that for $500 (1/49*$500=$10.20) and the other amounts. The expected payout is somewhat less when more individuals select a given $ amount as a drawing is made among all individuals that selected one of the 7 dollar amounts above. Again, the dollar amount is determined from drawing one dollar ticket among the 49 total tickets for each dollar amount.*

Part B: If you were required to pay to attend today's training, what is the most you would be Willing-To-Pay? $ ______________________

Module 3: Site-specific items: small-town / beginning-farmer

(These items were administered at the small-town / beginning-farmer site.)

F1. *Prior to* attending these Beginning Farmer classes, what were your intentions regarding developing a business plan? *(select one)* ☐ Definitely Yes ☐ Definitely No ☐ Maybe ☐ Never Considered it

F2. *After* attending these Beginning Farmer classes, what are your intentions regarding developing a business plan? *(select one)* ☐ Definitely Yes ☐ Definitely No ☐ Maybe ☐ Never Considered it

F3. Have you, or do you plan to, use any of the UA record-keeping binders or pocket notebooks provided? *(select one)* ☐ Have already used both ☐ Utilized 1 of the 2 provided ☐ Plan to use at least one of these ☐ Plan to use both of these ☐ Currently use other record-keeping tools ☐ Do not plan to utilize

F4. What are your intentions for growing and marketing specialty crops? *(select one)* ☐ Currently sell ☐ Currently give away ☐ Definitely yes ☐ Probably ☐ Grow only for home consumption ☐ Other

F5. Do you currently have land to farm? *(select all that apply)* ☐ Yes, own land ☐ Yes, lease land ☐ No

F6. Knowledge self-assessment (small-town / beginning-farmer).

Please provide two responses for each statement below. First, circle the answer that describes your knowledge **BEFORE** today's training on the following subjects. Then in the section labeled "**NOW or AFTER**," circle the answer that describes your knowledge **AFTER** attending the classes noted.

MY UNDERSTANDING										
Please describe your understanding or level of knowledge of the following:	BEFORE attending the class					NOW or AFTER attending a class				
	Poor	Fair	Good	Very Good	Excellent	Poor	Fair	Good	Very Good	Excellent
[topic 1]	1	2	3	4	5	1	2	3	4	5
[topic 2]	1	2	3	4	5	1	2	3	4	5
[topic 3]	1	2	3	4	5	1	2	3	4	5
[topic 4]	1	2	3	4	5	1	2	3	4	5
[topic 5]	1	2	3	4	5	1	2	3	4	5
[topic 6]	1	2	3	4	5	1	2	3	4	5
Overall Class Series Rating						1	2	3	4	5

Note: Specific class topics varied by training series; the original forms printed the relevant class dates and topics for each series.
Note: The small-town / beginning-farmer form did not include the travel-cost-coverage question (see Module 4). In the analysis, travel cost at this site was treated as self-covered.

Module 4: Site-specific items: rural-area / beginning-rancher

(These items were administered at the rural-area / beginning-rancher site.)

R1. How was your travel cost covered today? *(select all that apply)* ☐ By myself ☐ By other family members ☐ By an organization or employer

R2. Knowledge self-assessment (rural-area / beginning-rancher). *Scale and Before/After columns as in F6.*

Please provide two responses for each statement below. First, circle the answer that describes your knowledge **BEFORE** today's training on the following subjects. Then in the section labeled "**NOW or AFTER**," circle the answer that describes your knowledge **AFTER** attending the classes noted.

MY UNDERSTANDING										
Please describe your understanding or level of knowledge of the following:	BEFORE attending the class					NOW or AFTER attending a class				
	Poor	Fair	Good	Very Good	Excellent	Poor	Fair	Good	Very Good	Excellent
[topic 1]	1	2	3	4	5	1	2	3	4	5
[topic 2]	1	2	3	4	5	1	2	3	4	5
[topic 3]	1	2	3	4	5	1	2	3	4	5
[topic 4]	1	2	3	4	5	1	2	3	4	5
[topic 5]	1	2	3	4	5	1	2	3	4	5
[topic 6]	1	2	3	4	5	1	2	3	4	5
Overall Rating of Today's Training						1	2	3	4	5
