# Supplementary material for: Valuing education for limited-resource audiences using a willingness to accept experiment
Source: PLoS One. 2026 Sep 16;21(9):e0348858. doi: 10.1371/journal.pone.0348858 (PMC13581028; doi:10.1371/journal.pone.0348858)
Supplement: Supplementary file 2 [file pone.0348858.s002.pdf]

S1 Table. Robustness check: perceived-benefit variables added to the fully specified OLS and ordered-probit models (Models 2.5 and 3.5).

	OLS		Oprobit	
	Model 2.5	Model 2.5 and added variables	Model 3.5	Model 3.5 and added variables
Explanatory Variables				
Ln(WTP)	67.798 *	65.022*	0.490 **	0.467 *
	(33.729)	(34.628)	(0.242)	(0.248)
DBegFarm	107.930 *	107.070 *	0.717 *	0.726 *
	(61.552)	(61.077)	(0.419)	(0.414)
AvgKnowBef	40.524	47.065	0.390 *	0.434 *
	(29.16)	(37.095)	(0.213)	(0.262)
AvgKnowAft	-99.006 **	-98.96 **	-0.849 ***	-0.839 ***
	(37.423)	(42.57)	(0.301)	(0.317)
TravC	-0.011	0.0152	0.0007	0.0009
	(0.278)	(0.276)	(0.002)	(0.002)
Education	3.028	1.317	-0.020	-0.032
	(10.923)	(11.08)	(0.076)	(0.078)
Male	53.485	57.89	0.195	0.226
	(47.004)	(49.06)	(0.316)	(0.328)
Age	0.365	0.565	-0.0008	0.001
	(1.700)	(1.718)	(0.011)	(0.011)
PerceivedBenefit		19.16		0.094
		(62.07)		(0.405)
SolidifiedCommit		-31.57		-0.247
		(49.95)		(0.358)
Constant	176.7	189.3		
	(265.8)	(262.3)		
Observations	58	58	58	58
R^2	0.260	0.267		
Adjusted R^2	0.139	0.111		
AIC	770.5	774.0	183.4	186.8
BIC	789.0	796.7	212.2	219.8

Note: Robust standard errors are in parentheses. *,** and *** indicate 10%, 5%, and 1% statistical significance levels, respectively.
