# Supplementary material for: Valuing education for limited-resource audiences using a willingness to accept experiment
Source: PLoS One. 2026 Sep 16;21(9):e0348858. doi: 10.1371/journal.pone.0348858 (PMC13581028; doi:10.1371/journal.pone.0348858)
Supplement: Supplementary file 3 [file pone.0348858.s003.pdf]

S2 Table. Distributions of collected perception items included in the public de-identified dataset.

Responses to the two participant-perception items collected by the questionnaire and retained in the public de-identified dataset. These items were not reported in the original submission and are the measures used in the sensitivity analysis reported in S1 Table.

Response category	n	%
Panel A. Perceived benefit of the session (PerceivedBenefit). Questionnaire item: "Do you believe today's classes/training would or will improve your success?"		
Definitely	77	74.8
Probably	23	22.3
Maybe	3	2.9
No difference	0	0.0
**Total valid responses**	**103**	**100.0**
Item not answered	11	—
Panel B. Influence on future direction (SolidifiedCommit). Questionnaire item: "How have today's classes/training influenced your direction regarding being a future farmer/rancher?"		
Solidified current commitment to farming or ranching	54	52.9
Definitely like to start some day	36	35.3
No change / undecided	11	10.8
Convinced me not to farm or ranch	1	1.0
**Total valid responses**	**102**	**100.0**
Item not answered	12	—

Notes. Percentages are of valid (non-missing) responses to each item and may be reproduced from the public de-identified dataset (variables class_improve_reality and class_influence_future).

Denominator. The deposited dataset contains 118 questionnaire records; the 114 records with non-missing one-way travel distance constitute the analysis sample used throughout the manuscript, and the distributions above are computed on those 114 records. Item-level missingness accounts for the remaining cases (11 and 12, respectively).

Coding used in the sensitivity analysis (S1 Table). PerceivedBenefit equals one if the participant answered "Definitely" and zero otherwise; SolidifiedCommit equals one if the participant answered "Solidified current commitment to farming or ranching" and zero otherwise.

The response option "No difference" was offered for the first item but was not selected by any respondent. Questionnaire item wording is reproduced from the blank instrument provided as S1 Appendix.
